# Metazoan Maelstrom is an RNA-binding protein that has evolved from an ancient nuclease active in protists

**DOI:** 10.1261/rna.049437.114

**Published:** 2015-05

**Authors:** Kuan-Ming Chen, Edgar Campbell, Radha Raman Pandey, Zhaolin Yang, Andrew A. McCarthy, Ramesh S. Pillai

**Affiliations:** 1 European Molecular Biology Laboratory, Grenoble Outstation, 38042 Grenoble Cedex 9, France; 2 Unit for Virus Host-Cell Interactions, University Grenoble Alpes-EMBL-CNRS, 38042 Grenoble Cedex 9, France

**Keywords:** MAEL, piRNA, EHI_192630, ribonuclease, *Bombyx*, *E. histolytica*, Piwi

## Abstract

Piwi-interacting RNAs (piRNAs) guide Piwi argonautes to their transposon targets for silencing. The highly conserved protein Maelstrom is linked to both piRNA biogenesis and effector roles in this pathway. One defining feature of Maelstrom is the predicted MAEL domain of unknown molecular function. Here, we present the first crystal structure of the MAEL domain from *Bombyx* Maelstrom, which reveals a nuclease fold. The overall architecture resembles that found in Mg^2+^- or Mn^2+^-dependent DEDD nucleases, but a clear distinguishing feature is the presence of a structural Zn^2+^ ion coordinated by the conserved ECHC residues. Strikingly, metazoan Maelstrom orthologs across the animal kingdom lack the catalytic DEDD residues, and as we show for *Bombyx* Maelstrom are inactive as nucleases. However, a MAEL domain-containing protein from amoeba having both sequence motifs (DEDD and ECHC) is robustly active as an exoribonuclease. Finally, we show that the MAEL domain of *Bombyx* Maelstrom displays a strong affinity for single-stranded RNAs. Our studies suggest that the ancient MAEL nuclease domain evolved to function as an RNA-binding module in metazoan Maelstrom.

## INTRODUCTION

Mobile genetic elements like transposons have successfully colonized a substantial portion of eukaryotic genomes. Approximately 45% of the human genome can be directly traced back to their origin from transposable elements. While most of these are broken inactive repetitive sequences, some are full-length copies that encode proteins facilitating their mobility within the genome. Their insertion into new locations can cause mutations and promote ectopic recombination events that threaten genome integrity. Germlines are tasked with the job of faithfully transmitting genetic information from one generation to the next and this makes them particularly sensitive to any transposition events. Chromatin and DNA modifications stably silence transposons in the genome, but epigenetic upheavals that accompany germ cell development have the potential to release them from this repression (Malone and Hannon 2009). To guard against transposon activity, animal gonads have evolved a dedicated small RNA-based transposon defense system that uses Argonaute proteins of the Piwi clade and their associated ∼24–31 nucleotide (nt) Piwi-interacting RNAs (piRNAs) (Ghildiyal and Zamore 2009).

Consistent with their role in transposon silencing, piRNAs bear sequence complementarity to host transposon sequences. This allows piRNAs to guide Piwi proteins to their nucleic acid targets. In the cytoplasm, Piwi endonucleases (slicers) destroy transposon transcripts, while nuclear Piwi complexes specify chromatin or DNA modifications to prevent transcription from target transposon genomic loci. Information on which genomic elements are to be targeted is contained in discrete genomic loci called piRNA clusters. These clusters are ∼50–150 kb in length and hold a diverse array of transposon fragments representing all transposable elements in the host genome. Long single-stranded cluster transcripts are converted into tens of thousands of primary piRNAs that provide germ cells with an arsenal to target every transposon in the genome. Germ cells also possess a mechanism to amplify those silencing molecules that are most useful at any given point in time. The initiator of such an amplification process is the endonucleolytic cleavage of a target transposon transcript by a Piwi protein. After this, one of the Piwi-generated cleavage fragments gets loaded into a new Piwi protein to mature as a (sense-oriented) secondary piRNA. Secondary piRNA biogenesis in the *Bombyx mori* (Silkworm) ovary-derived BmN4 cell cultures (Kawaoka et al. 2009) is mediated by an ATP-dependent piRNA Amplifier complex assembled on transposon targets by the DEAD box RNA helicase Vasa. This complex facilitates transfer of a cleavage fragment between the two Piwi proteins (from Siwi to Ago3) expressed in these cells (Xiol et al. 2014). Such secondary piRNAs then guide cleavage of complementary sequences (for example, in long cluster transcripts) to generate the exact same antisense piRNA that initiated the whole process. This adaptive piRNA amplification process called the ping-pong cycle generates more silencing molecules every time target transposons are cleaved (Brennecke et al. 2007). Most known piRNA biogenesis factors are enriched in perinuclear, cytoplasmic granules called nuage, and these are also thought to be major centers of piRNA action (Lim and Kai 2007). So piRNA precursors and transposon targets have to meet up with the pathway factors within these germline granules.

Genetic screens and biochemical studies have uncovered a wealth of factors that are necessary for piRNA biogenesis and transposon silencing in both the fly and mouse models. *Maelstrom* (Findley et al. 2003) was originally identified as a member of the *spindle*-class mutants that display female sterility and patterning defects in the developing fly oocyte. Maelstrom is a steady-state resident of the nuage, but it is unique in being a nucleo-cytoplasmic shuttling protein (Findley et al. 2003). Subsequent studies linked Maelstrom to retrotransposon control in the fly germline (Lim and Kai 2007), a role that is also conserved in mice (Soper et al. 2008). Given the role in transposon control and subcellular localization with other piRNA biogenesis factors in the fly and mouse nuage, Maelstrom was linked to a possible role in the piRNA pathway.

Transcriptional silencing in the *Drosophila* ovarian cells is mediated by the prototypic Piwi clade member, Piwi, which promotes deposition of H3K9me3 on transposon sequences. Analysis of piRNA levels in fly *mael* mutant ovaries revealed no impact on piRNA biogenesis (Sienski et al. 2012). Given that the nuclear function of Piwi is independent of Maelstrom, the protein was proposed to play a silencing effector role in an unknown step downstream from chromatin methylation (Sienski et al. 2012). Any such role linked to chromatin may not be conserved as Maelstrom is also expressed in the *Bombyx* BmN4 cells that clearly lack a nuclear Piwi pathway (Xiol et al. 2014). Furthermore, Maelstrom is shown to associate with components of the microtubule-organizing center (MTOC) (Sato et al. 2011) and transcriptionally repress *microRNA-7* in fly ovaries (Pek et al. 2009). Finally, in the mouse male germline Maelstrom binds primary piRNA precursors and Piwi proteins, and *mael* mutants display reduced piRNA levels, indicating a role in piRNA biogenesis (Castaneda et al. 2014). All these genetic studies point to multiple roles for a protein that still remains poorly understood biochemically.

## RESULTS

### Crystal structure of the MAEL domain of *Bombyx* Maelstrom

**FIGURE 1.**
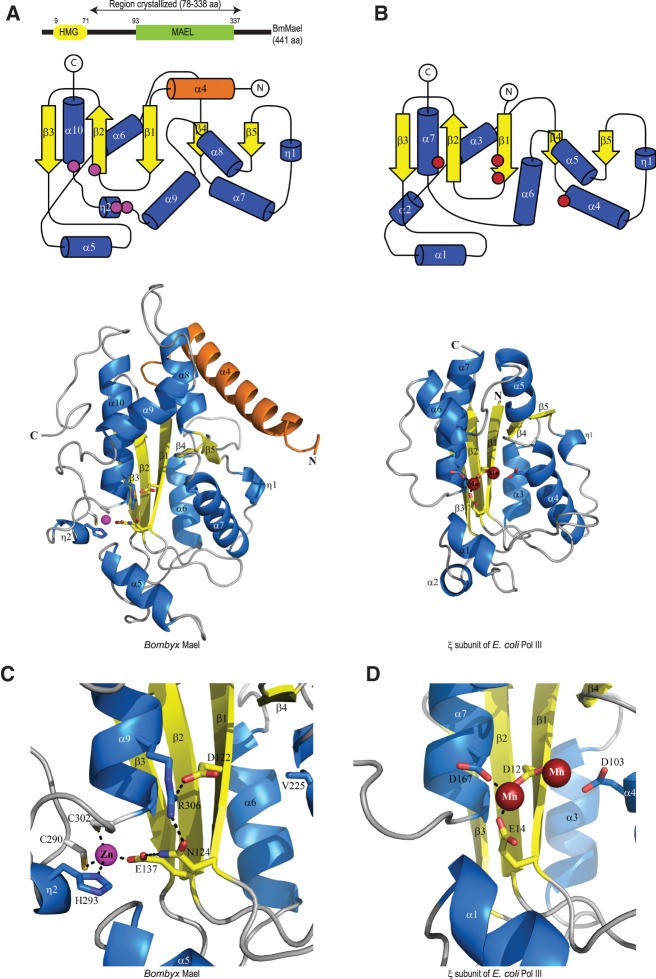
The MAEL domain of *Bombyx* Maelstrom reveals a nuclease fold. (*A*) A cartoon showing the domain architecture of *Bombyx mori* Maelstrom (BmMael) indicating the High Mobility Group (HMG) box and the MAEL domain specific to Maelstrom. Structural topology representation of the crystal structure shown *below*: Helices as cylinders (blue or orange) and β-strands as arrows (yellow) are shown. Crystal structure of the MAEL domain from BmMael. The amino-terminal linker helix in BmMael connecting the MAEL domain to HMG is colored in orange. (*B*) Structure of the DnaQ nuclease domain from the ε subunit of *E. coli* Pol III (PDB: 1J53). (*C*) Zoomed view highlighting the residues coordinating the structural Zn^2+^ ion in MAEL. Note the absence of catalytic residues (DEDD) in the MAEL domain that are essential for nuclease activity in (*D*) the DnaQ domain. Dotted black lines indicate hydrogen bond interactions.

Maelstrom is composed of an amino-terminal High Mobility Group (HMG) box and a predicted carboxy-terminal MAEL domain that is a signature feature of the protein across the animal kingdom (Fig. 1A; Zhang et al. 2008). The HMG box is shown to confer DNA-binding to proteins harboring it in other contexts (Bianchi and Agresti 2005). In contrast, information on the biochemical properties and structure of the MAEL domain is not available. To explore this, we crystallized a fragment of *Bombyx* Maelstrom (BmMael) (see Materials and Methods) containing the MAEL domain. This reveals an α/β structure (Fig. 1A) composed of an amino-terminal linker helix followed by the MAEL domain. The linker helix α_4_ (colored in orange) connects the MAEL domain to the amino-terminal HMG box, which in human Maelstrom is made of three helices (RIKEN Structural Genomics/Proteomics Initiative). The MAEL domain (Fig. 1A) is composed of a central β-sheet composed of five strands (β_1_–β_5_), where β_2_ is antiparallel to the others. The β-sheet is flanked on one face by three helices (α_8_–α_10_) and on the other by two helices (α_6_ and α_7_). The bottom of the sheet is capped by helix α_5_. This basic configuration is strikingly similar to that found in DEDD family nucleases, as represented by the ε-subunit of *Escherichia coli* polymerase III (PDB: 1J53) (Hamdan et al. 2002), which can be superimposed with a RMSD of 1.6 Å for 74 of a possible 174 Cα atoms (Fig. 1B).

Despite this overall resemblance between MAEL and DEDD nuclease domains, one striking difference is the presence of a Zn^2+^ ion in the MAEL structure that bridges the bottom of the β-sheet and the loop connecting α_9_ and α_10_, which encompasses η_2_ (Fig. 1A,C). The Zn^2+^ ion is tetrahedrally coordinated by E137 from β_2_, H293 from η_2_, and residues C290 and C302 from loops (Fig. 1C). The fact that the Zn^2+^ ion was captured from the bacterial culture media, and not added during initial crystallization trials, points to its paramount importance for the structural integrity of the MAEL domain. Mutational studies in *Drosophila* Maelstrom also support such a structural role for the Zn^2+^ ion, as single point mutations in the conserved ECHC motif of *Drosophila* Maelstrom (E131A and H291A), identical to E137 and H293 in *Bombyx*, abrogate its ability to complement mutant flies for transposon silencing and fertility (Sienski et al. 2012). Interestingly, these *Drosophila* mutants fail to enter the nucleus, pointing to a structural collapse of the protein (Sienski et al. 2012). Thus, our structure reveals that the MAEL domain takes up a unique nuclease fold embellished with a new Zn binding motif (ECHC).

### Metazoan MAEL domain is inactive while that of protists have ribonuclease activity

A closer comparison of the *Bombyx* MAEL domain with the DEDD nuclease domain in *E. coli* Pol III reveals that the catalytic residues (D12, E14, D103, and D167) essential for coordinating two Mn^2+^ ions in the bacterial enzyme are not conserved in the MAEL domain (Fig. 1D). Only the first residue of the DEDD motif (D122 from β_1_) is conserved in the *Bombyx* MAEL domain. All the other residues are replaced by those (N124 and R306) that preclude any ability in coordinating divalent metal ions. Intriguingly, an extensive hydrogen bonding network links these inactive residues in the catalytic pocket: N124 from β_1_ forms hydrogen bonds (dotted black line in figure) with both E137 of the ECHC motif and R306 from α_9_, while R306 has an additional interaction with D122 from β_1_ (Fig. 1C). Furthermore, the α_1_ in the Pol III structure is more proximal to the catalytic residues than α_5_ in the MAEL structure, making the catalytic pocket in the DEDD nuclease marginally more compact when compared with MAEL.

**FIGURE 2.**
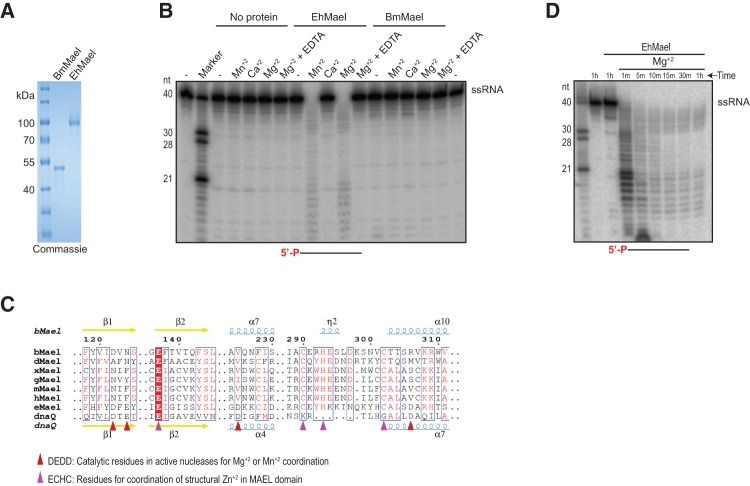
*Bombyx* Maelstrom is inactive as a nuclease but activity is retained in its protist ortholog. (*A*) Instant Blue staining of recombinant BmMael and *E. histolytica* MAEL domain (EhMael) proteins used for nuclease assay. (*B*) Nuclease activity of proteins tested on a single-stranded RNA (ssRNA). Reactions were incubated for 1 h with indicated proteins in the absence (−) or presence of different metal ions. In reactions containing EhMael and appropriate metal ions (Mn^2+^ and Mg^2+^) the input ssRNA was completely degraded and appears as a RNA ladder. Note the complete absence of such an activity with BmMael. (*C*) Selected regions from a sequence alignment of the MAEL domain from *Bombyx* (b), *Drosophila* (d), *Xenopus* (x), *Gallus* (g), mouse (m), human (h), and *E. histolytica* (e) Maelstrom proteins. DnaQ is the nuclease domain from the ε subunit of *E. coli* Pol III. The secondary structure elements: α-helices, 3_10_ helices (η), and β-strands are shown *above* (for BmMael) and *below* (for DnaQ from Pol III; PDB: 1J53) the alignment. The arrowheads indicate conserved residues required for catalytic activity (red) and Zn^2+^ coordination (magenta). (*D*) A time-course of nuclease activity with EhMael. Time in minutes (min) or hours (h) is indicated.

To directly test the catalytic potential of the *Bombyx* MAEL domain, we incubated the recombinant full-length BmMael protein with a 5′-end labeled single-stranded RNA (ssRNA) (Fig. 2A). As shown in Figure 2B, over 1 h of incubation at 27°C did not yield any cleavage products. Addition of three different metal ions did not change this situation. The MAEL domain is also detected in protists (Zhang et al. 2008), where the catalytic residues DEDD are retained along with the MAEL-specific ECHC motif (Fig. 2C). To test whether the protist MAEL domain harbors nuclease activity, we produced a recombinant version of the MAEL domain-containing protein (EHI_192630) from the human parasitic protozoan *Entamoeba histolytica* virulent strain HM-1:IMSS (Fig. 2A). Incubation of the protein (EhMael) alone with a ssRNA did not result in its degradation, while addition of Mg^2+^ or Mn^2+^ resulted in complete elimination of the input RNA and appearance of a ladder of RNA degradation fragments (Fig. 2B,D). Specificity of the reaction is indicated by the fact that the nuclease activity was inhibited by incubation with the Mg^2+^-chelating agent EDTA. Use of Ca^2+^ did not result in any activity (Fig. 2B). These results suggest that the MAEL domain confers ribonuclease activity in protists where the catalytic DEDD residues are preserved, while metazoan Maelstrom proteins (including *Bombyx* and *Drosophila*) lack these (Fig. 2C), and are hence inactive.

### Metazoan MAEL domain functions as an RNA-binding module

**FIGURE 3.**
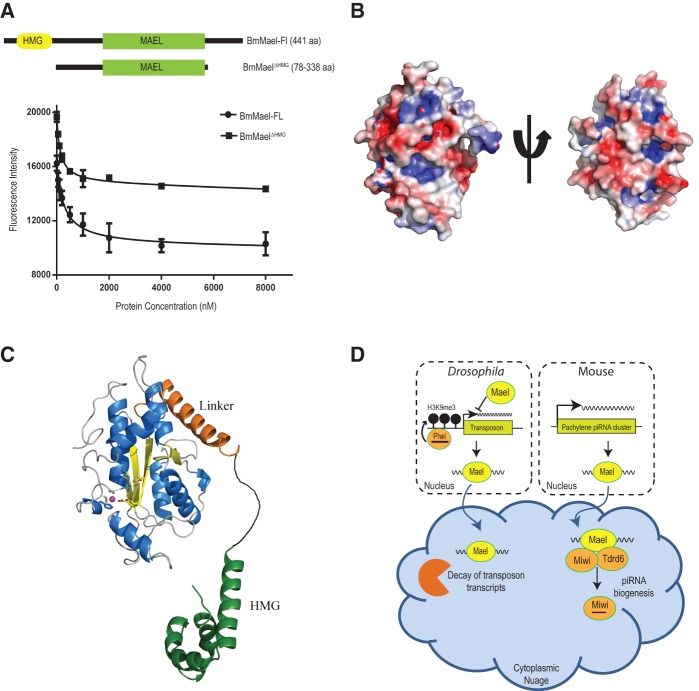
*Bombyx* Maelstrom is an RNA-binding protein. (*A*) Cartoon showing BmMael constructs used for RNA-binding studies. Fluorescence emission measurements (three independent experiments) using a RNA molecule labeled with a fluorophore and the BmMael proteins are indicated. (*B*) Surface charge representation of MAEL domain from BmMael contoured from −6 kTe^−1^ (red) to +6 kTe^−1^ (blue) generated using PyMOL APBS tools. (*C*) A composite structural model of Maelstrom created using the MAEL domain of *Bombyx* Mael and the HMG box domain of human Mael (PDB: 2CTO). (*D*) A model for Maelstrom's role in the piRNA pathway in the female *Drosophila* and male mouse germline.

Since the *Bombyx* MAEL domain has the core nuclease architecture conserved we tested if it might allow binding to RNA substrates. We examined this with a fluorescently labeled ssRNA and measured decreased fluorescence intensity as readout of protein binding (Fig. 3A). This indicated 216.7 ± 75.57 nM binding affinity for full-length BmMael. Interestingly, the construct lacking the HMG box (containing only the linker helix plus the MAEL domain) also showed robust affinity for RNA (129.9 ± 18.86 nM). Examination of charge distribution on the MAEL domain did not indicate a clear positively charged surface for RNA binding (Fig. 3B). It is therefore likely that the RNA is contacted at a location close to the inactive catalytic pocket. The HMG box may also contribute to nucleic acid binding, and mutants lacking this domain are nonfunctional in vivo (Sienski et al. 2012).

## CONCLUSIONS

Taken together, our study provides structural and biochemical insights into the MAEL domain that is a signature feature of Maelstrom. We show that the MAEL domain is active as a nuclease in protists, while inactive in metazoans, as in the case of *Bombyx*. We show that the *Bombyx* MAEL can function as an RNA-binding module and that this activity might be further supported by the HMG box, a known nucleic acid binding domain (Fig. 3C). Since *Drosophila* and mouse Maelstrom proteins also lack the DEDD catalytic residues (Fig. 2C), we propose that the functions genetically attributed to the protein can only be explained by this RNA-binding activity. In the case of *Drosophia* germline, Maelstrom might bind transposon transcripts arising from genomic loci undergoing Piwi-mediated transcriptional gene silencing (TGS) (Sienski et al. 2012) and evacuate them to cytoplasmic nuage, where it is delivered to general RNA degradation enzymes for post-transcriptional gene silencing (*trans*-PTGS) (Fig. 3D). Similarly, in the case of the mouse male germline, Maelstrom is shown to directly bind pachytene piRNA precursors (Castaneda et al. 2014). Since mouse Maelstrom is shown to interact with piRNA pathway factors, it might deliver the precursor RNAs to the processing machinery for generation of pachytene piRNAs (Castaneda et al. 2014). In the case of BmN4 cells, mass spectrometry analysis of HA–Maelstrom complexes also identified associations with piRNA biogenesis factors (data not shown). Thus, the ancient MAEL nuclease domain, after acquiring mutations, was requisitioned into an RNA-binding role in the metazoan piRNA pathway. It is also possible that the MAEL domain in Maelstrom may provide a protein interaction surface for other factors that facilitate its function in the piRNA pathway.

## MATERIALS AND METHODS

### Clones and constructs

The complementary DNA (cDNA) encoding *Bombyx mori* (Silkworm) Maelstrom (Mael) was isolated from total RNA of BmN4 ovarian cell cultures by reverse-transcription PCR (RT-PCR). The cDNA for MAEL domain-containing protein (EHI_192630) from the human parasitic protozoan *Entamoeba histolytica* virulent strain HM-1:IMSS was chemically synthesized (Shanghai ShineGene Molecular Biotech, Inc.) using available sequence information (XM_648799.2). The full-length proteins were expressed as amino-terminal 6xHis-Sumo tag fusions in either *E. coli* (for BmMael) or in *Spodoptera frugiperda* 21 (Sf21) insect cell cultures (for EhMael) using recombinant baculovirus preparations.

The full-length BmMael protein was subjected to limited proteolysis using trypsin protease and the boundaries of stable fragments were defined by peptide mass fingerprinting using MALDI (Proteomics Core Facility, EMBL). All BmMael constructs were expressed from the bacterial expression vector (pETM-11Sumo vector; EMBL Protein Expression and Purification Core Facility) as 6xHis-SUMO-TEVsite-fusions: 28–350 aa, 78–350 aa, 115–350 aa, 78–338 aa. The construct carrying the amino-terminal linker helix and carboxy-terminal MAEL domain (78–338 aa) was crystallized and used in this study.

The full-length EhMael was cloned into the pACEBac2Sumo acceptor vector for expression as a 6xHis-SUMO fusion in the *Spodoptera frugiperda* 21 (*Sf*21) insect cell line.

### Maelstrom purification

The *Bombyx* Mael constructs were transformed into the *E. coli* Rosetta strain and grown overnight at 16°C. Cells were lysed (25 mM Tris [pH 8.0], 150 mM NaCl, 20 mM Imidazole, 0.5% Tween 20, 5mM β-mercaptoethanol, and Protease Inhibitor [Roche]) and His-tagged proteins were purified over Ni-NTA beads. After removal of the 6xHis-Sumo tag by overnight TEV cleavage, the untagged recombinant protein was further purified over an ion-exchange column: Q-column (GE Health) for BmMael (78–338 aa) or SP-column for full-length BmMael. Fractions containing the recombinant proteins were further purified by size-exclusion chromatography (S75 or S200). Highly pure fractions from this last step were used for crystallography, biophysical measurements, and nuclease assays. The 6xHis-Sumo-EhMael was similarly purified, except that the tag was not removed.

### Crystallization, data collection, and structure solution of *Bombyx* Maelstrom

Crystals of native and SeMet substituted protein were grown in hanging drops at 4°C using solutions containing 25 mM HEPES (pH 7.0), 150 mM NaCl, 50 µM ZnCl_2_, and 10 mM DTT. Crystals of the native protein appeared as small needles after 7 d and were further improved using micro and macro seeding techniques. The SeMet protein typically produced rhombohedral shaped crystals. The native and SeMet crystals were flash frozen at 100 K after transferring them to identical crystallization conditions containing 20% glycerol. The SeMet protein crystallized in space group R32 and contained three molecules in the asymmetric unit. A highly redundant 3.4 Å SeMet anomalous data set was collected at the peak and inflection of the Se-Met signal, as measured by X-ray fluorescence for experimental phasing on ID23-1 (Nurizzo et al. 2006) at the European Synchrotron Radiation Facility (ESRF). The native protein crystallized in space group P21, contained four molecules in the asymmetric unit and a data set to 2.4 Å was obtained using the helical data collection procedure on ID29 at the ESRF (de Sanctis et al. 2012). Integration and scaling was carried out with the XDS suite (Kabsch 2010) and SCALA (Evans 2006), respectively. A summary of the data statistics is given in Table 1. The resolution cut off was chosen to ensure a *I*/σ(*I*) > 1.0 and a CC(1/2) > 50%. We note that sedimentation velocity experiments reveal that Maelstrom does not oligomerize and is monomeric in solution (data not shown).

**TABLE 1.**
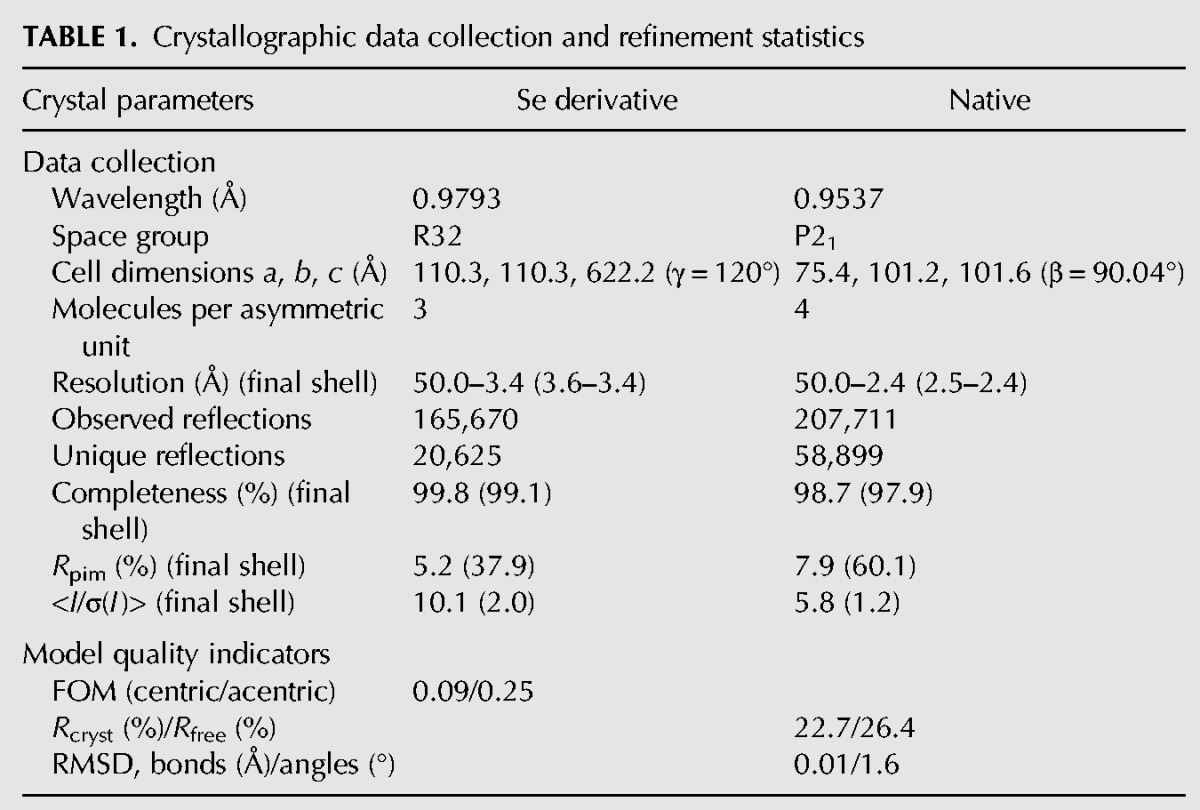
Crystallographic data collection and refinement statistics

For the structural solution 14 Se-Met sites were located on the basis of their anomalous differences using SHELXC/D/E (Sheldrick 2010). These sites were refined and experimental phases to 3.4 Å were calculated using the multiple anomalous dispersion (MAD) procedure in autoSHARP. These phases were further improved using Crank2 (Skubak and Pannu 2013), incorporating model building and refinement with Buccaneer (Cowtan 2006) and REFMAC5 (Murshudov et al. 2011). The initial model produced was positioned in the native data set with Phaser (McCoy et al. 2007). The native data set was determined to have pseudomerohedral twinning so all subsequent refinement cycles were performed using REFMAC (Murshudov et al. 2011) or Phenix (Afonine et al. 2012) with NCS restraints and twinning options enabled. Model building was carried out with Coot (Emsley et al. 2010) and the stereochemical quality of the protein molecules were validated with Molprobity (Chen et al. 2010). All the crystallographic information is summarized in Table 1 and the crystallographic coordinates have been deposited within the Protein Data Bank (PDB: 5AF0).

### Nuclease assays

Highly purified protein preparations (Ni-affinity column, ion exchange, and gel-filtration) were incubated at 1.0 μM concentrations with a 5′-end radiolabeled single-stranded 40-nucleotide RNA in the reaction buffer (25 mM Tris–HCl [pH 7.5], 150 mM NaCl, 2 mM DTT) for 1 h (1 h) at 27°C. When required, divalent metal ions (2.5 mM CaCl_2_, or 2.5 mM MnCl_2_, or 10 mM MgCl_2_) or the Mg-chelating agent EDTA (100 mM) were added prior to addition of the radiolabeled RNA. For time-course experiments, reactions were stopped after several minutes (1–30 min) by addition of phenol–chloroform and extracted RNA was examined by 15% urea-PAGE analysis.

### Biophysical studies

Sedimentation velocity experiments (Plateforme Biophysique, Partnership for Structural Biology) were done on an analytical ultracentrifuge XLI (Beckman Coulter) with a rotor speed of 42,000 rpm, at 4°C, using a rotor Anti-60, and double-sector cells of optical path length 12 mm with Sapphire windows. Acquisitions were made using absorbance at 280 nm and interference optics. The reference is the buffer (25 mM MES, 150 mM NaCl, and 5 mM DTT [pH 6.0]) used for the sample. The analysis was done with the SEDFIT software, version 14.4fb and Gussi 1.0.8d.

The RNA-binding studies were carried out using fluorescence spectroscopy. Fluorescence emission measurements of 50 nM fluorescently labeled RNA (5′-FAM-Ex-5-UAUACCUCUGCUUCUGCU-3′) with increasing concentrations of BmMael constructs were monitored at 25°C (excitation at 495 nm and emission at 515 nm) in the reaction buffer containing 25 mM MES (pH 6.0), 150 mM NaCl, 5 mM DTT, and 50 µM ZnCl_2_. The equilibrium dissociation constant *K*_D_ was determined by fitting the data using a nonlinear regression analysis (one site total binding) in Prism software.

## DATA DEPOSITION

Structural coordinates are deposited within the Protein Data Bank under code no. 5AF0.

## NOTE ADDED IN PROOF

We note that the Lassa virus nucleoprotein (PDB: 4GV9), which is an active 3′-5′ exoribonuclease, has both the ECHC and the DEDD motifs required for Zn and Mn/Mg coordination, respectively. This again highlights the importance of DEDD motif for nuclease activity, as we showed here for the MAEL-domain protein from amoeba.
